# Improved computations for relationship inference using low-coverage sequencing data

**DOI:** 10.1186/s12859-023-05217-z

**Published:** 2023-03-09

**Authors:** Petter Mostad, Andreas Tillmar, Daniel Kling

**Affiliations:** 1 Mathematical Sciences, Chalmers University of Technology and the University of Gothenburg, Göteborg, Sweden; 2 Department of Forensic Genetics and Toxicology, National Board of Forensic Medicine, Linköping, Sweden; 3 Department of Biomedical and Clinical Sciences, Linköping University, Linköping, Sweden; 4 Department of Forensic Sciences, Oslo University Hospital, Oslo, Norway; 5 Biostatistics (BIAS), Norwegian University of Life Sciences, Ås, Norway

**Keywords:** LcNGS, Pedigree inference, Bayesian

## Abstract

Pedigree inference, for example determining whether two persons are second cousins or unrelated, can be done by comparing their genotypes at a selection of genetic markers. When the data for one or more of the persons is from low-coverage next generation sequencing (lcNGS), currently available computational methods either ignore genetic linkage or do not take advantage of the probabilistic nature of lcNGS data, relying instead on first estimating the genotype. We provide a method and software (see familias.name/lcNGS) bridging the above gap. Simulations indicate how our results are considerably more accurate compared to some previously available alternatives. Our method, utilizing a version of the Lander-Green algorithm, uses a group of symmetries to speed up calculations. This group may be of further interest in other calculations involving linked loci.

**Supplementary Information:**

The online version contains supplementary material available at 10.1186/s12859-023-05217-z.

## Introduction

Genetic marker data has been used to resolve questions about relationships for several decades [1–4]. The core idea is to leverage how differences in the Mendelian inheritance of alleles through alternative pedigrees will result in probabilistic differences in how genotypes of tested persons relate. In forensic genetics, traditional software such as Familias [5, 6] and DNAview [7] can be used to efficiently compute the likelihood ratio for two or more hypotheses about possible pedigrees, when genetic markers are unlinked. In op. cit., versions of the Elston Stewart algorithm [8] are used which efficiently handle large pedigrees, modelling mutations throughout the pedigree. In medical genetics and linkage analysis, software such as Merlin [9] implements a version of the Lander-Green algorithm [10] efficiently handling linked markers on smaller pedigrees.

Advances in genetic sequencing (e.g. next-generation sequencing, NGS) [11, 12] have decreased the cost of whole genome sequencing (WGS) considerably and also brought about several new panels targeting a greater number of genetic markers. Tillmar et al. published a panel of genetic markers with relevance to forensic and population genetics, encompassing roughly 4000 kinship informative single nucleotide polymorphism (SNP) markers [13]. The panel can be analyzed on low-end benchtop genetic sequencers such as the MiSeq (Illumina) or the Ion S5 (Thermo Scientific) instruments. In medical and population studies, high density SNP microarrys are commonly used, where data for hundreds of thousands of markers are simultaneously genotyped. However, for samples with low quality and quantity, such as forensic grade or archaeological samples, genetic sequencing is often a superior choice [14] yielding more information to be used in the ensuing evaluations.

A standard procedure, having access to good quality and high-coverage next generation sequencing data, is to apply a threshold-based approach to call the genotypes, and then proceed as above, even if the called genotypes may then either contain many errors, or a lot of missing data, or both [15–17]. In this paper, we focus on situations where at least some of the data is of low-coverage next generation sequencing (lcNGS) type and/or DNA samples contain very little or degraded DNA and is derived from a sequencing analysis. There is no generally agreed definition of low-coverage in this context. Average sample coverage of 30X or more can mean high coverage, while 1X or less is clearly low-coverage. In this paper, we use 10X or below to mean low-coverage sequence data. Due to an expected high proportion of no calls/missing data using the standard procedure for such datasets, we explore a more probabilistic approach where we establish a likelihood for all possible genotypes of the tested persons given the observed data, and use such genotype likelihoods directly in calculations relating to hypotheses about relatedness.

The program NgsRelate [18], widely used to infer relatedness from lcNGS data, uses a maximum likelihood approach to find the most likely set of Jacquard coefficients between all pairs of individuals in a given data set, although the method does not consider genetic linkage between markers. On the other hand, the program Merlin [9] computes likelihoods for genotype data given different hypotheses about relationships accounting for genetic linkage and a simple model for genotyping errors but fails to implement a complete model for data from sequencing.

We will show that our approach can give more reliable likelihood results than using algorithms that first determine a genotype, or ignore linkage. Further, we show that our approach can yield useful results even for lcNGS data with low coverage, or when using samples based on very little DNA, so that many heterozygote balances are off. We believe our approach is unique in combining a probabilistic observational data model for sequencing read data with an inheritance model accounting for genetic linkage for hypotheses about relationship concerning two or more individuals. An implementation is available from https://familias.name/lcNGS.

## Materials and methods

In Sects. 2.2 through 2.6 we will define a method that, given lcNGS data for two or more individuals and two alternative pedigrees relating them, computes a likelihood ratio. Combining this ratio with a prior odds yields a posterior odds which may be used to choose between the pedigrees. To assess such a method, the best alternative is of course to apply it and competing methods to large numbers of cases where the true pedigree is known in each case. Such comparisons are limited by the availability of data.

An alternative, used in this paper, is to compare competing methods on simulated data. The assessment is then divided into two tasks: Showing that the simulation model yields data that is realistic in relevant ways, and comparing methods on the simulated data. The simulation model used in this paper uses population models and inheritance models presented in Sects. 2.3 and 2.4, where the inheritance model includes linkage (i.e., effects of crossovers inside the considered pedigree). When two loci are strongly linked, their alleles will often be inherited together, as haplotypes, through the pedigree, thus strongly influencing the information about the pedigree contained in the data. This motivates why data simulation should contain linkage.

The population model includes important standard features such as kinship, however, it does *not* include linkage disequilibrium (LD, i.e., effects of crossovers outside of the considered pedigree). This means that the effect of LD on competing methods is not assessed. Current methods for handling LD include grouping markers together [9] or using an multiorder Markov chain [19]. Both ideas may be possible to combine with our approach. We have chosen to defer treatment of LD to a later paper.

Section 2.2 presents the observational model we use to simulate lcNGS data from simulated genotypes. This is a simplified model simulating only counts of reads at each locus. Section 3.1 contains a small study and an argument why we believe this observational model captures features of lcNGS data essential for relationship inference, in particular when one or more of the samples are based on small amounts of DNA.

Our likelihood method for pedigree inference uses exactly the same likelihood as the one used in data simulation. In any simulation study, when simulation is done using a particular probability distribution, it will be optimal to use the same distribution for likelihood computations. What our study illustrates is the size of the performance reduction when using a likelihood method that ignores linkage or the uncertainty in genotypes that is inherent in lcNGS data. Finally, we compare our approach with NgsRelate [18] which uses a maximum likelihood procedure to find the most likely Jacquard coefficients. NgsRelate does not account for genetic linkage between the included genetic markers.

### Model overview

We assume we have data concerning *N* autosomal loci for *T* tested persons. For each considered pedigree we would like to compute the probability of the observed data given the pedigree. We model this probability using a population model including an $F_{st}$ adjustment [20] but no linkage disequilibrium (LD), an inheritance model including a Poisson crossover model but no mutations, and an observational model featuring reads that are sampled from true alleles with some possibilities for errors. The text below is formulated in terms of markers with at most four alleles, which is the nature of SNPs. However, the model is not per se restricted to tetra-allelic data.

### Observational model

At a given genetic locus for a tested person, we assume the data consists of a vector $c=(c_1,c_2,c_3,c_4)$ of counts of reads corresponding to nucleotides A, C, G, T, respectively. Assume the true genotype at this locus is $g=(g_1,g_2)$, coded as two indices between 1 and 4. We describe the relationship between *c* and *g* as a result of two separate stochastic events: First, the proportion *q* of DNA segments after PCR that are based on $g_1$ among those based on either $g_1$ or $g_2$ is modelled as $q=k/m$ where $k\sim {\text {Binomial}}(m,1/2)$. Here *m* is an integer parameter connected to the sample, representing the approximate number of DNA templates from the sample that end up founding PCR amplicons for this locus. For high *m*, we have $q\approx 1/2$, while for lower *m*, *q* can be close or even equal to 0 or 1, meaning that one of the alleles failed to be picked up in the PCR process.

Writing $C=c_1+c_2+c_3+c_4$ and $\beta =(\beta _1,\beta _2,\beta _3,\beta _4)$, we model $c\mid q, g \sim {\text {Multinomial}}\left( C, \beta \right)$ where for $i=1,2,3,4$,

1 $$\begin{aligned} \beta _i = \left( 1-e\right) (qI(g_1=i) + (1-q)I(g_2=i)) + \frac{e}{4} \end{aligned}$$

where *e* is a small positive model parameter^1^ relating to e.g. sequencing or mapping errors. In other words, for each observation of a read, there is a small probability *e* that it is in fact unrelated to the underlying genotypes, and the probability is then 1/4 for reporting each genotype. With probability $1-e$, the read is based on $g_1$ with probability *q* and on $g_2$ with probability $1-q$. Putting the two stochastic events together we get that

2 $$\begin{aligned} {\text {Pr}}(c\mid g) = \sum _{k=0}^m{\text {Pr}}(c\mid q=k/m, g)\left( {\begin{array}{c}m\\ k\end{array}}\right) 2^{-m}. \end{aligned}$$

In Sect. 3.1 we compare calculated probabilities from the model above with real data to argue that the two parameters *m* and *e* in our model can capture the most important features of variability in observational data. We note that low quality and quantity DNA samples can be modelled with a low *m* (sometimes in the range 5–10), since few and damaged DNA molecules is directly correlated to a low *m*. On the other hand, *e*, usually attributed to sequencing or mapping errors, is generally low (quite close to zero) with modern sequencing and bioinformatic tools [21].

To make likelihood computations for case sample data with the model above, a user has to provide information about the parameters *m* and *e*. One possibility is to use the results of Sect. 3.1 to select values. Alternatively a user may provide priors: If *M* possible values $m_1,m_2,\dots ,m_M$ with probabilities $p_1,p_2,\dots ,p_M$ approximately describes prior knowledge about the parameter *m*, and similarly *E* possible values $e_1,\dots ,e_E$ with probabilities $q_1,\dots ,q_E$ describes a prior for the parameter *e*, then we may compute

3 $$\begin{aligned} {\text {Pr}}(\text {data}\mid \text {pedigree}) = \sum _{i=1}^M\sum _{j=1}^E{\text {Pr}}(\text {data}\mid \text {pedigree},m_i,e_j)p_iq_j. \end{aligned}$$

### Population model

We assume population independence between different loci, i.e. there is no association (linkage disequilibrium, LD) between alleles at different loci. At a locus, assume there are *F* “founding alleles”, so that the remaining alleles in the pedigree are inherited from these. We provide a stochastic model for the vector $h=(h_1,h_2,h_3,h_4)$ of counts of how many of the *F* founding alleles are of each of the four possible types. Assume the population frequencies of the four possible alleles are $f=(f_1,f_2,f_3,f_4)$. We then model

4 $$\begin{aligned} h\mid f,\theta ,\gamma \sim {\text {Dirichlet-Multinomial}}\left( F, \left( 1/\theta -1\right) \left[ f(1-\gamma )+\gamma {\overline{f}}\right] \right) . \end{aligned}$$

Here, $\theta$ is the kinship parameter (population fixation parameter) $F_{st}$ [20] while $\gamma$ is a small positive number and ${\overline{f}}$ is a vector with general probabilities for observing A, C, G, or T for any of the markers. Note that with this model, after having observed *k* alleles of type *i* and $s-k$ alleles of other types, the probability for observing another allele of type *i* is

5 $$\begin{aligned} \frac{k + (1/\theta -1)(f_i(1-\gamma )+\gamma {\overline{f}}_i)}{s + 1/\theta -1 } = \frac{\theta k + (1-\theta )(f_i(1-\gamma )+\gamma {\overline{f}}_i)}{1 + \theta (s-1)}. \end{aligned}$$

Note also that Eq. 5 reduces to the traditional $F_{st}$ formula [20] when $\gamma =0$.

To motivate why one might use a number larger than zero for $\gamma$, consider the following example: We compute the likelihood ratio comparing the alternatives of two persons being unrelated or half-siblings using data at a single locus. Assume data for both persons contains 50 reads indicating nucleotide G, while the population frequency of G is zero. With $\gamma =0$, all reads for G will be attributed to read errors (which may seem unreasonable) and the likelihood ratio would be approximately 1. With $\gamma$ some small positive number, the likelihood ratio in favour of unrelatedness would instead be approximately equal to the probability of observing a second G allele among the founder alleles of the case data after having observed a first G allele, which in our model would be

6 $$\begin{aligned} \theta + (1-\theta )\gamma {\overline{f}}_3. \end{aligned}$$

Finally, note that our model can be interpreted as using frequencies from a database of size $(1/\theta -1)(1-\gamma )$ and a pseudo-count vector $(1/\theta -1)\gamma {\overline{f}}$.

### Inheritance model

Given a pedigree consisting of *K* parent–child relationships. Consider the set ${{\mathcal {A}}}$ consisting of vectors *r* of length *K* where each component is either 0 or 1, with 0 indicating that the child in the corresponding relationship has inherited the parent’s maternal allele, while 1 indicates inheritance of the paternal allele. We call such a vector an inheritance pattern. Each value of *r* organizes the alleles of the persons in the pedigree into subsets of alleles that must be identical as long as we disregard mutations, which is reasonable to do for SNPs. Restricting ourselves to the typed persons, we represent such a partition as a vector of length 2*T* of subset indices, with each of the *T* pairs representing the maternal and paternal alleles of a person. We enumerate the subsets using consecutive integers starting from zero. For any two subsets, if there exists one or more persons in which alleles from exactly one of the subsets occur, consider the first person, in a fixed ordering of the persons, in which this happens, and the subset with an allele in this person. This subset will then be indexed with a lower integer compared to the other subset. Finally, for each pair of integers representing the alleles of a single person, if the first integer is larger than the second, we switch the two integers. This creates a unique code for each partitioning of unordered pairs of alleles: We call this an IBD code.

As an example, consider a nephew and his paternal uncle. We get two possible IBD codes (0, 1, 2, 3) and (0, 1, 0, 2). As the pedigree may be defined by $K=5$ relationships, we have that the corresponding *r* vector has $2^5=32$ possible values. Each of these values map to one of the IBD codes above. If we assign equal probability to each of the possible *r* vectors, the induced probabilities on the two IBD codes above are both 0.5.

Let us now write $r_i$ for the inheritance pattern at locus *i*. Let *h*(*r*) denote the IBD code for an $r\in {{\mathcal {A}}}$. Given the IBD code for a locus and the observational and population models above, we may compute the probability of the observed data at the locus by conditioning on and summing over all possible combinations of alleles for the subsets indicated by the IBD code. Write $L_i(h(r_i))$ for the probability^2^ of the data at locus *i*. The functions $L_i$ are determined by our observational and population models. The complete model probability can now be written

7 $$\begin{aligned} {\text {Pr}}(\text {data}\mid \text {pedigree}) = \sum _{(r_1,\dots ,r_N)}{\text {Pr}}(r_1,\dots ,r_N)\prod _{i=1}^NL_i(h(r_i)) \end{aligned}$$

where we sum over all possible inheritance patterns for all loci.

It remains to specify the joint probability model for the vectors $r_1,r_2,\dots ,r_N$. To simplify we assume a Markov model so that each $r_{i+1}$ is independent of $r_1,\dots ,r_{i-1}$ given $r_i$. Specifically, we assume there is a given probability $p_i$ for an odd number of crossovers between locus *i* and $i+1$ independently for all relationships defining the pedigree. This yields the conditional probabilities

8 $$\begin{aligned} T_i(r_i,r_{i+1}) \overset{\text {def}}{=}{\text {Pr}}(r_{i+1}\mid r_i) = \prod _{j=1}^Kp_i^{I(r_{ij}\ne r_{i+1,j})} (1-p_i)^{I(r_{ij}=r_{i+1,j})}, \end{aligned}$$

where we write $r_i=(r_{i1},r_{i2},\dots ,r_{iK})$ and $r_{i+1}=(r_{i+1,1},r_{i+1,2},\dots ,r_{i+1,K})$. The Markov assumption together with a uniform probability on $r_1$ now yields a joint probability model for $r_1,r_2,\dots ,r_N$.

### A possible computational algorithm

The Markov assumption above makes it possible to use an iterative algorithm to compute the value of Eq. 7, in fact, a version of the Lander-Green algorithm [10]. Specifically, let us write $d_i$ for the data at locus *i* and suppress the pedigree from the notation. For $i=1,\dots ,N$ and all values of $r_i$, we may compute

9 $$\begin{aligned} {\text {Pr}}(d_1,\dots ,d_i,r_i) = {\text {Pr}}(d_1,\dots ,d_{i-1},r_i) {\text {Pr}}(d_i\mid r_i) \end{aligned}$$

and (for $$i<N$$)

10 $$\begin{aligned} {\text {Pr}}(d_1,\dots ,d_i,r_{i+1}) =\sum _{r_i} {\text {Pr}}(d_1,\dots ,d_i,r_i) {\text {Pr}}(r_{i+1}\mid r_i). \end{aligned}$$

Noting that ${\text {Pr}}(d_i\mid r_i)=L_i(h(r_i))$, that ${\text {Pr}}(r_{i+1}\mid r_i)=T_i(r_i, r_{i+1})$, and that

11 $$\begin{aligned} {\text {Pr}}(\text {data}\mid \text {pedigree}) = {\text {Pr}}(d_1,\dots ,d_N) = \sum _{r_N}{\text {Pr}}(d_1,\dots ,d_N,r_N), \end{aligned}$$

we obtain the following algorithm:

Initialize a vector *z* of length $2^K$ with values $1/2^K$, so that it represents the prior probability distribution on $r_1$. Then, for each locus $i=1,\dots ,N$:

1. Compute a vector of length $2^K$ by computing $L_i(h(r_i))$ for all possible values of $r_i$. Multiply it term-wise with *z* to get a vector representing ${\text {Pr}}(d_1,\dots ,d_i,r_i)$ for all possible values of $r_i$.
2. If $i<N$, compute a matrix M of size $2^K\times 2^K$ representing $T_i(r_i,r_{i+1})$ for all possible values of $r_i$ and $r_{i+1}$. Then set *z* equal to the matrix product *zM*, so that *z* now represents ${\text {Pr}}(d_1,\dots ,d_i,r_{i+1})$.

Finally, sum the elements of *z* to obtain the probability we wanted to compute.

The computed number will in real examples be extremely small, so that one needs to compute its logarithm to avoid numerical underflow. In practice we re-scale the values in *z* in every loop above, storing separately the logarithm of a common factor.

### An improved algorithm using symmetries

An important problem with the algorithm above is that $2^K$ can be a large number even for fairly small *K*, so that the $(2^K\times 2^K)$ matrix *M* representing $T_i(r_i,r_{i+1})$ can become too large to handle. However, it turns out that in practice the vector *z* will contain many repeated values. This opens up the possibility of using a “compressed” matrix $M^*$ for computations.

Specifically, consider a subdivision

12 $$\begin{aligned} {{\mathcal {A}}}= & {} \{v_{11},v_{12},\dots ,v_{1n_1}\}\cup \{v_{21},v_{22},\dots ,v_{2n_2}\}\cup \dots \nonumber \\{} & {} \dots \cup \{v_{J1},v_{J2},\dots ,v_{Jn_J}\} \end{aligned}$$

of the set of inheritance patterns ${{\mathcal {A}}}$ into disjoint subsets where for all *v* and $v^*$ in a common subset there exists a permutation *g* on ${{\mathcal {A}}}$ so that (1) for all $r\in {{\mathcal {A}}}$ $h(g(r))=h(r)$, (2) for all $r,r'\in {{\mathcal {A}}}$ *g*(*r*) and $g(r')$ differ in the same number of components as *r* and $r'$, and (3) $g(v)=v^*$. Then we will show in the Additional file 1 Appendix that

13 $$\begin{aligned} {\text {Pr}}(d_1,\dots ,d_i,r_i=v) = {\text {Pr}}(d_1,\dots ,d_i,r_i=v^*) \end{aligned}$$

and that for all $i=1,\dots ,N-1$ and $k=1,\dots ,J$

14 $$\begin{aligned} {\text {Pr}}(d_1,\dots ,d_i,r_{i+1}=v_{k1}) =\sum _{j=1}^J{\text {Pr}}(d_1,\dots ,d_i,r_i=v_{j1}) \left( \sum _{s=1}^{n_j}T_i(v_{js},v_{k1})\right) \end{aligned}$$

yielding the following improved algorithm:

Initialize a vector *z* of length *J* with values $1/2^K$. Then, for $i=1,\dots ,N$:

1. Compute a vector of length *J* by computing $L_i(h(v_{j1}))$ for $j=1,\dots ,J$. Multiply it term-wise with *z* to get a vector representing ${\text {Pr}}(d_1,\dots ,d_i,r_i=v_{j1})$ for $j=1,\dots ,J$.
2. If $i<N$, compute a matrix $M^*$ of size $J\times J$ representing $\sum _{s=1}^{n_j}T_i(v_{js},v_{k1})$ for $j,k=1,\dots ,J$. Then set *z* equal to the matrix product $zM^*$, so that *z* now represents ${\text {Pr}}(d_1,\dots ,d_i,r_{i+1}=v_{j1})$ for $j=1,\dots ,J$.

Finally, sum the elements of *z* to obtain the probability we wanted to compute.

To use this algorithm, one needs to find a subdivision like the one described above. See the Appendix for an algorithm that derives the optimal subdivision. We also need to compute the matrix $M^*$ for each *i*. In the Appendix we show that

15 $$\begin{aligned} \sum _{s=1}^{n_j}T_i(v_{js},v_{k1}) = \sum _{r=0}^KA_r(v_{j1},v_{k1})p_i^r(1-p_i)^{K-r} \end{aligned}$$

where

16 $$\begin{aligned} A_r(v_{j1},v_{k1}) = \#\left\{ v_{js}: v_{js} \text { and} v_{k1} \text { differ at} r \text { locations}\right\} . \end{aligned}$$

In other words, the entries of the matrix is a polynomial in $p_i$, with integer coefficients found by counting the differences between inheritance pattern representatives. Note that, to any pedigree there is associated a unique such matrix of polynomials. As they may play a fundamental role in pedigree computations using Markov-modelled linkage, we believe such matrices should be studied further. We look at a number of examples in Sect. 3.2.

### Data, and simulation and comparison procedure

**Table 1:** Drop-in and drop-out measurements for 12 samples.

Sample	Input(ng)	Mean coverage	Drop-in	Drop-out
1	20	657	0.0195	0
2	20	40	0.0227	0
3	20	20	0.0237	0.0040
4	20	8	0.0243	0.0537
5	20	4	0.0220	0.2597
6	20	2	0.0167	0.6160
7	20	1	0.0067	0.9223
8	1	105	0.0179	0
9	0.25	59	0.0143	0.0196
10	0.125	35	0.0129	0.0822
11	0.031	12	0.0092	0.5558
12	0.015	9	0.0091	0.7461

Drop-in is measured as the proportion of reads indicating an allele that is not present in the genotype the read reports for. Drop-out is measured as the proportion of heterozygous loci where there are reads for only one allele type

The assessment of our observation model in Sect. 3.1 uses the Coriell sample NA12878, a genomic reference material (Coriell Institute) sequenced in Tillmar et al. [13] on a Illumina MiSeq instrument. Results for samples with varying amounts of DNA and varying allelic depths are shown in Table 1.

The simlation uses 3929 SNPs from Tillmar et al. [13], describing a SNP panel with autosomal SNPs evenly spread across the chromosomes. Genetic positions are downloaded from Ruther’s repository [22]. From Tillmar et al, we further use genotype data for the Coriell sample NA12878 to obtain coverage statistics. Allele frequencies are extracted for individuals with European ancestry (CEU) from the 1000 Genomes project [23]. We generate founder alleles through the population model in Sect. 2.3 with $\theta =0.01$ and $\gamma =0.001$. We continue to drop alleles through the pedigree using the inheritance model in Sect. 2.4, with crossover probabilities derived from the genetic positions alluded to above. Next, to mimic low coverage data (lcNGS) based on reduced-quality samples, we use the model in Sect. 2.2 with $m=10$ and $e=0.02$ to generate sequence read data. The allelic depths are drawn independently for each locus using a discretized Gamma distribution, first with expectation 10 and standard deviation 2 for Figs. 2 and 3 and then with expectation 3 and standard deviation 1 for Figs. 4 and 5.

**Fig. 1:**
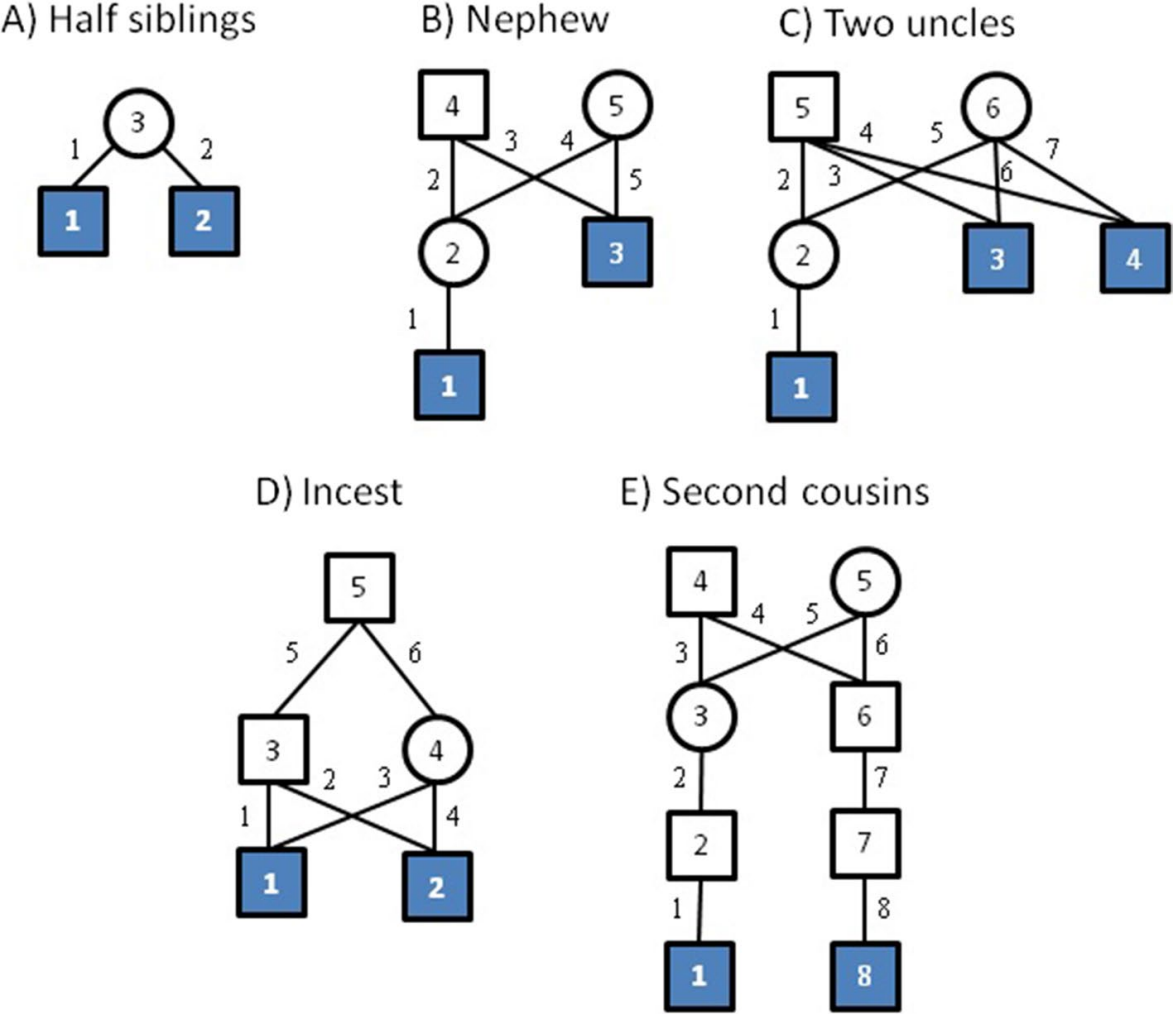
Our five example pedigrees. For each we indicate the numbering of the persons, the numbering of the parent–child relationships and which persons are tested (filled symbols)

In the simulation study in Sect. 3.3 we focus on whether two persons are second cousins (see Fig. 1) or unrelated. For each relationship 1000 cases are simulated and a Likelihood Ratio in favour of relatedness is computed using three different methods: Our proposed method, an amended version where linkage is ignored, and an amended version where genotypes are called. To make it optimally competitive, the calling algorithm uses the same likelihood as in our model, combining it with prior probabilities for genotypes based on allele frequencies and selecting the genotype that maximizes the resulting posterior. In other words, the called genotype is the one that maximizes the product of the population frequency of the genotype and the likelihood of the data given the genotype, where the likelihood is computed as in Sect. 2.2.

For each simulated case we also estimate Jacquard coefficients using NgsRelate [18]. We use VCF-files as input, with PL-fields derived from the same data likelihoods we use in our proposed method. The Euclidean distances from the estimated point $k=(k_0,k_1,k_2)$ of non-inbred coefficients to corresponding points representing the second cousin relationship or unrelatedness are computed. Comparing the difference in distances to a cutoff value yields a classification of cases into related or unrelated. Varying the cutoff value yields receiver operating characteristic (ROC) curves seen in Figs. 3 and 5. For comparison, the figures also show results for other methods, converted to ROC curves using the LR as cutoff.

### Implementation

R-code with our algorithm is available at https://familias.name/lcNGS. To validate the correctness of our implementation, we used the software Merlin [9] which has been widely used to compute likelihoods for pedigrees and genetic data. We constructed input files for Merlin using data for a range of cases with simulated data for the SNP markers published in Tillmar et al [13]. Likelihoods were computed in Merlin and in the R script alluded to above implementing our model and subsequently compared.

Our R implementation of the pedigree preprocessing step had running times of 2–3 s for a pedigree of second cousins on a standard 2.6 GHz laptop. A complete example computation provided at the link above, involving preprocessing and comparing 5 pedigrees, had a running time of about a minute.

## Results

Our method for computing pedigree likelihoods can be described as consisting of two steps: First, the pedigree is pre-processed, in the sense that its IBD codes and symmetries are computed, as well as the matrices of Eq. 16. Such results may be of interest in their own right, and Sect. 3.2 provides a number of examples. In the second step, the probability of sample data given alternative pedigrees is computed, as in Sect. 2.6.

Section 3.3 contains our simulation study where our proposed method is compared with various alternatives. We also look at the extent to which our method can yield correct conclusions even with very low allelic depths. However, we start with Sect. 3.1, exploring whether our observational model appears appropriate for lcNGS data.

### Behavior of observational model

Discrepancies between true genotypes and bioinformatically called genotypes based on lcNGS data can take various forms. For instance, alleles may drop in, i.e. reads for alleles not in the genotype are present. Main causes of drop-in are sequencing errors, mapping errors and contamination. Assuming a contamination is discovered with other tools, we focus on modelling the occurrence of sequencing and mapping errors.

For the test samples listed in Table 1 (see Sect. 2.7 for details) drop-in rates for bioinformatically called genotypes vary hugely, from zero (sample 1) to 0.1057 (sample 5), as such rates are very influenced by allelic depth. However, if we instead count the rate at which a read represents an allele not present in its underlying genotype, we get much more stable numbers, as seen in Table 1. For homozygous loci, our observational model predicts that each read has an independent probability 3*e*/4 of being of a type not present in the genotype; for heterozygous loci the probability is 2*e*/4. As the genotype on which the samples are based has a 57% proportion of homozygous loci, we expect a drop-in rate of 0.6425*e* as defined above. Comparing with the table, we see that we can use values of *e* around 0.02 (or in the interval 0.01$-$0.04) for samples of the type listed in the table. When different setups of the sequencing machinery or different settings of the bioinformatic pipeline are used, other values for *e* may be more appropriate. See, e.g., [24] and [25] for some relevant studies.

The main challenge for lcNGS data is generally allelic drop-out, meaning in practice that reads of only one allele are observed for a true heterozygous genotype. Homozygote genotypes can naturally drop out, but will be interpreted as missing data which is not as problematic. At low allelic depths, drop-out can occur by chance during the target enrichment or sequencing process. Additionally, with low input amounts of DNA an allele can drop out completely even before the target enrichment, or the balance of two alleles will be skewed during enrichment, increasing the probability of drop-out at the sequencing stage. Table 1 lists the proportion of heterozygous loci in our samples where reads for only one of the alleles are seen.

**Table 2:** Values for $a_{m,d}$ of Euqation 17, using $e=0.02$

$m\backslash d$	1	2	4	10	20	40	100
1	0.99	0.9703	0.9413	0.8597	0.7391	0.5463	0.2206
2	0.99	0.7302	0.5307	0.4307	0.3696	0.2732	0.1103
4	0.99	0.6101	0.3146	0.1327	0.0936	0.0683	0.0276
5	0.99	0.5861	0.2733	0.0864	0.0489	0.0342	0.0138
10	0.99	0.5381	0.1939	0.0243	0.0041	0.0012	0.0004

We model drop-out with the *m* parameter together with the Eqs. 1 and 2. At a heterozygous locus with allelic depth *d*, we get the probability

17 $$\begin{aligned} a_{m,d} = \sum _{k=0}^m\left( {\begin{array}{c}m\\ k\end{array}}\right) 2^{-m} \left[ \left( (1-e)\frac{k}{m}+\frac{e}{4}\right) ^d + \left( (1-e)\left( 1-\frac{k}{m}\right) +\frac{e}{4}\right) ^d\right] \end{aligned}$$

that all reads are of one of the true alleles. Table 2 lists this probability for various combinations of *m* and *d*, using $e=0.02$. We see that by using $m=10$ we can to some degree reproduce the behaviour of drop-out probabilities for samples 1 through 7. Samples 8 through 12 can be roughly represented using *m* values 10, 5, 4, 2, and 1, respectively. We see that the appropriate value for *m* decreases with the amount of DNA in the sample, in correspondence with our motivation for the observational model.

The comparisons above between actual observations and model predictions is quite rough, ignoring for example variability in allelic coverage between loci. The stochastic processes giving rise to lcNGS data are quite complex, and our simplistic observational model captures only a small part of this complexity. However, we argue that our model is still useful as a first approximation. The above is intended both as a motivation that our model is relevant, and a guide to understanding and setting the parameters *e* and *m*. A fuller statistical analysis comparing the model to alternatives is planned for a later paper.

### Preprocessing of pedigrees

In order to do likelihood computations involving linked loci, our method includes a pre-processing, which can be applied to any pedigree containing a list of tested persons. First, we compute a set of IBD codes, a set of representative inheritance patterns for each IBD code, and for all pairs of representatives a polynomial which may afterwards be used in likelihood computations. The theory for obtaining these results is given in Sects. 2.4 through 2.6 and in the Appendix. In the following section we show results from pre-processing the five example pedigrees illustrated in Fig. 1.

**Table 3:** IBD codes and representatives for the half sibling case

IBD codes	Prob	Represenatives
(0, 1, 0, 2)	0.5	1:(00)
(0, 1, 2, 3)	0.5	2:(10)

At any given autosomal locus, two half siblings have one allele IBD with probability 0.5, and zero alleles IBD with probability 0.5. In Table 3, the two corresponding IBD codes produced by our algorithm are shown, together with the two representatives for each IBD code. For example, the representative $r=(00)$ should be interpreted as person 1 inheriting the maternal allele from the mother, and person 2 also inheriting the maternal allele from the mother. For the representative $r=(10)$ person 1 inherits the paternal allele while person 2 inherits the maternal allele.

**Table 4:** Transition matrix for the half sibling case

*j*	*s*	$\sum _{k=1}^{n_j}T_i(v_{jk},v_s)$
1	1	$(1-p)^2 + p^2$
1	2	$2p(1-p)$
2	1	$2p(1-p)$
2	2	$(1-p)^2 + p^2$

There are 4 possible inheritance patterns in this case. However, because of symmetries, we need not compute explicitly with the patterns (01) and (11). Instead we use for computations a $(2\times 2)$ matrix with entries given in Table 4, where *p* is the probability on a single chromosome of an odd number of crossovers between loci *i* and $i+1$.

**Table 5:** The IBD codes and representatives for the nephew case

IBD codes	Prob	Represenatives
(0, 1, 0, 2)	0.5	1:(00000), 2:(01000)
(0, 1, 2, 3)	0.5	3:(11000), 4:(01010)

**Table 6:** Transition matrix for the nephew case

*j*	*s*	$\sum _{k=1}^{n_j}T_i(v_{jk},v_s)$
1	1	$(1-p)^5 + p(1-p)^4 + 2p^2(1-p)^3 + 2p^3(1-p)^2 + p^4(1-p) + p^5$
1	2	$2p(1-p)^4 + 2p^2(1-p)^3 + 2p^3(1-p)^2 + 2p^4(1-p)$
1	3	$2p(1-p)^4 + 2p^2(1-p)^3 + 2p^3(1-p)^2 + 2p^4(1-p)$
1	4	$4p^2(1-p)^3 + 4p^3(1-p)^2$
2	1	$2p(1-p)^4 + 2p^2(1-p)^3 + 2p^3(1-p)^2 + 2p^4(1-p)$
2	2	$(1-p)^5 + 2p^2(1-p)^3 + 4p^3(1-p)^2 + p^4(1-p)$
2	3	$p(1-p)^4 + 4p^2(1-p)^3 + 2p^3(1-p)^2 + p^5$
2	4	$2p(1-p)^4 + 2p^2(1-p)^3 + 2p^3(1-p)^2 + 2p^4(1-p)$
3	1	$2p(1-p)^4 + 2p^2(1-p)^3 + 2p^3(1-p)^2 + 2p^4(1-p)$
3	2	$p(1-p)^4 + 4p^2(1-p)^3 + 2p^3(1-p)^2 + p^5$
3	3	$(1-p)^5 + 2p^2(1-p)^3 + 4p^3(1-p)^2 + p^4(1-p)$
3	4	$2p(1-p)^4 + 2p^2(1-p)^3 + 2p^3(1-p)^2 + 2p^4(1-p)$
4	1	$4p^2(1-p)^3 + 4p^3(1-p)^2$
4	2	$2p(1-p)^4 + 2p^2(1-p)^3 + 2p^3(1-p)^2 + 2p^4(1-p)$
4	3	$2p(1-p)^4 + 2p^2(1-p)^3 + 2p^3(1-p)^2 + 2p^4(1-p)$
4	4	$(1-p)^5 + p(1-p)^4 + 2p^2(1-p)^3 + 2p^3(1-p)^2 + p^4(1-p) + p^5$

The second case involves an uncle/nephew relationship, which has the same IBD probabilities as half siblings. However, as well known [26, 27], pedigree computations using linked loci will be different. We now need to use the four representatives listed in Table 5. The entries of the $(4\times 4)$ *T* matrix are listed in Table 6.

**Table 7:** IBD codes and representatives for the two uncles case

IBD codes	Prob	Represenatives
(0, 1, 0, 2, 0, 2)	0.125	1:(0000000), 2:(0100000)
(0, 1, 2, 3, 2, 3)	0.125	3:(1100000), 8:(0100100)
(0, 1, 0, 2, 0, 3)	0.125	4:(0010000), 6:(0110000)
(0, 1, 2, 3, 0, 2)	0.125	5:(1010000), 10:(1010100)
(0, 1, 0, 2, 2, 3)	0.125	7:(1110000), 12:(1110100)
(0, 1, 2, 3, 2, 4)	0.125	9:(0010100), 11:(0110100)
(0, 1, 2, 3, 0, 4)	0.125	13:(0010010), 14:(1110010)
(0, 1, 0, 2, 3, 4)	0.125	15:(1110010), 16:(0110110)

Considering an extension of the previous example, assume there are three tested persons (see Fig. 1). The IBD codes become vectors of length 6. Table 7 lists the possible IBD codes and representative inheritance patterns, for this pedigree. Each IBD code has probability 1/8 at each locus. The *T* matrix becomes a $(16\times 16)$ matrix and is not listed.

**Table 8:** IBD codes and representatives for the incest case

IBD codes	Prob	Represenatives
(0, 1, 0, 1)	0.21875	1:(000000), 4:(110000), 11:(000010),
		14:(110010), 20:(111110)
(0, 1, 0, 2)	0.43750	2:(100000), 3:(010000), 6:(011000),
		12:(100010), 13:(010010),
		17:(111010), 19:(110110)
(0, 0, 1, 2)	0.03125	5:(101000)
(0, 0, 0, 1)	0.06250	7:(111000)
(0, 1, 2, 2)	0.03125	8:(010100)
(0, 1, 0, 0)	0.06250	9:(110100)
(0, 0, 0, 0)	0.03125	10:(111100)
(0, 1, 2, 3)	0.12500	15:(101010), 16:(011010), 18:(010110)

The fourth case describes an incestuous relationship between two half siblings. The IBD codes for their two children and representatives are listed in Table 8. The *T* matrix has size $(20\times 20)$ and is not listed.

**Table 9:** IBD codes and representatives for the second cousins case

IBD codes	Prob	Represenatives
(0, 1, 2, 3)	0.9375	1:(00000000), 3:(01000000), 4:(11000000),
		5:(00100000), 7:(01100000), 8:(11100000),
		9:(00001000), 10:(10001000), 11:(00101000),
		12:(10101000), 13:(01101000), 14:(11101000),
		15:(00000001), 16:(01000001), 17:(00100001),
		18:(01100001), 19:(00001001),
		20:(00101001), 21:(01101001)
(0, 1, 0, 2)	0.0625	2:(10000000), 6:(10100000)

The final case describes two second cousins, who have probability 0.0625 for sharing one allele IBD. The IBD codes and representatives are listed in Table 9. Note how computations may be done with 21 representatives, instead of with each of the $2^8=256$ inheritance patterns. This illustrates the power of using symmetries. Note how our algorithm finds more symmetries than those obtained by permuting the paternal and maternal alleles of founders or by switching the untyped parents.

### Simulation results

**Fig. 2:**
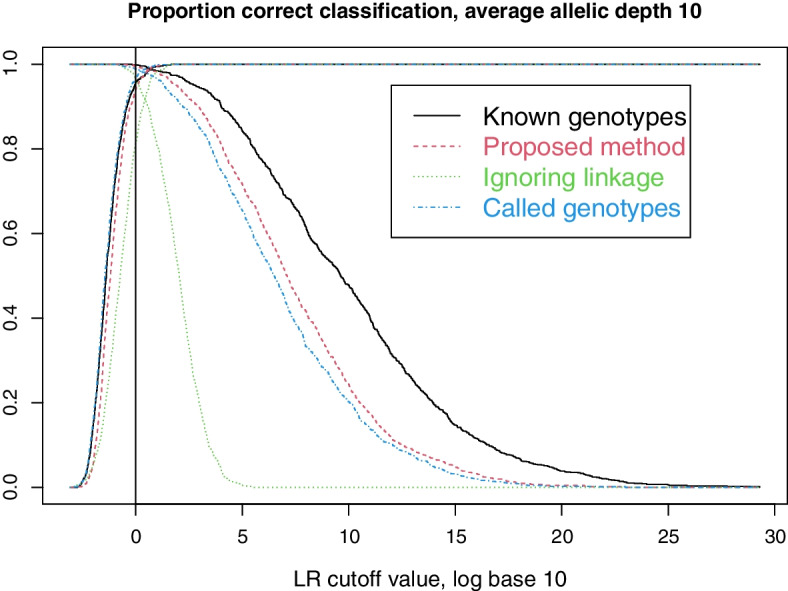
Decreasing (resp. increasing) lines show proportion of cases with (resp. without) relationship correctly classified

As described in Sect. 2.7 we simulated lcNGS data for 1000 cases where two persons are second cousins, and 1000 cases where they are unrelated. We then computed Likelihood Ratios in favour of relatedness using our proposed method, our proposed method with called genotypes, and our proposed method disregarding linkage. For the cases where the persons are related, Fig. 2 shows the proportion of LRs above the cutoff value given on the x-axis. Similarly, the proportion of LRs below the cutoff is shown for cases where the persons are unrelated.

**Fig. 3:**
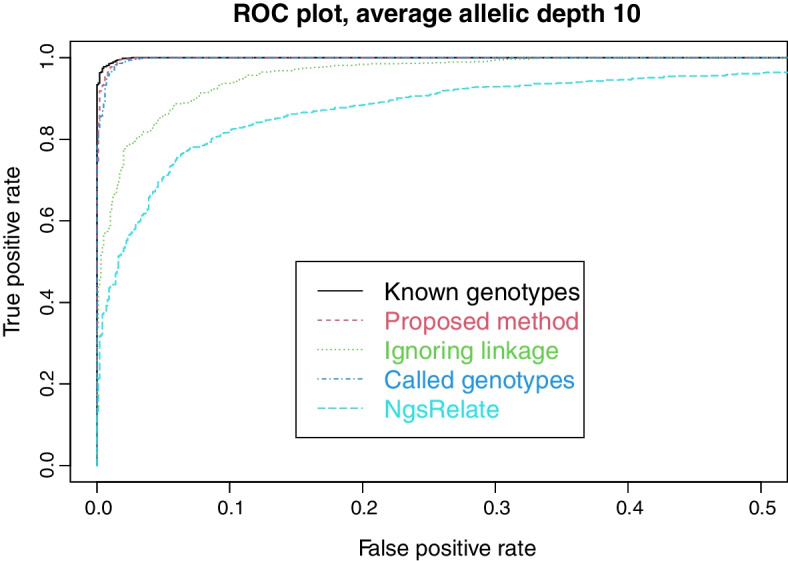
False positive and true positive rates for various methods, as the cutoff value changes

We see that most cases can be correctly resolved with the FORCE panel of SNPs and perfect knowledge about the genotypes, corroborating the results in Tillmar et al [13]. Also, the power to solve cases is only slightly reduced when using lcNGS data with average allelic depth 10 together with our proposed method. When the method is amended by calling genotypes, there is a small reduction in power, while there is a large reduction in power when the method is amended to ignored linkage. The same conclusions can be drawn from Fig. 3, which also includes results from NgsRelate. We see that using maximum likelihood estimates of Jacquard coefficients gives considerably less power to solve cases.

**Fig. 4:**
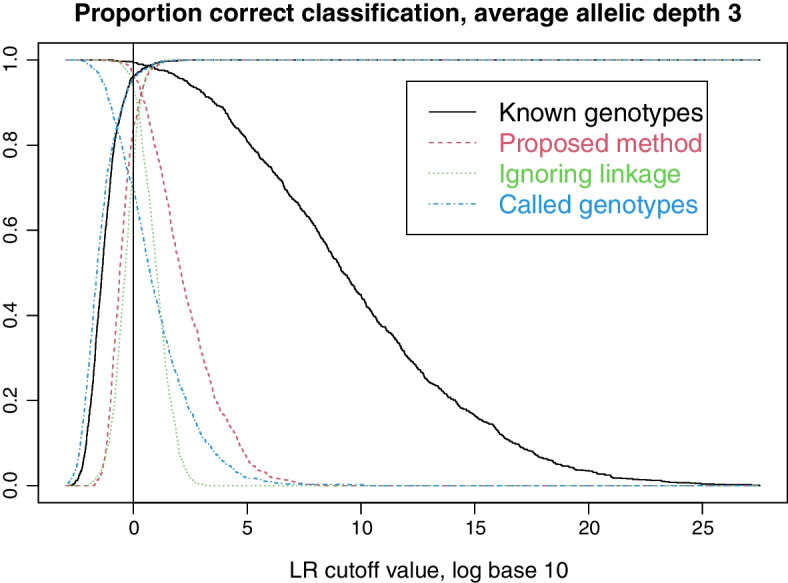
Decreasing (resp. increasing) lines show proportion of cases with (resp. without) relationship correctly classified

**Fig. 5:**
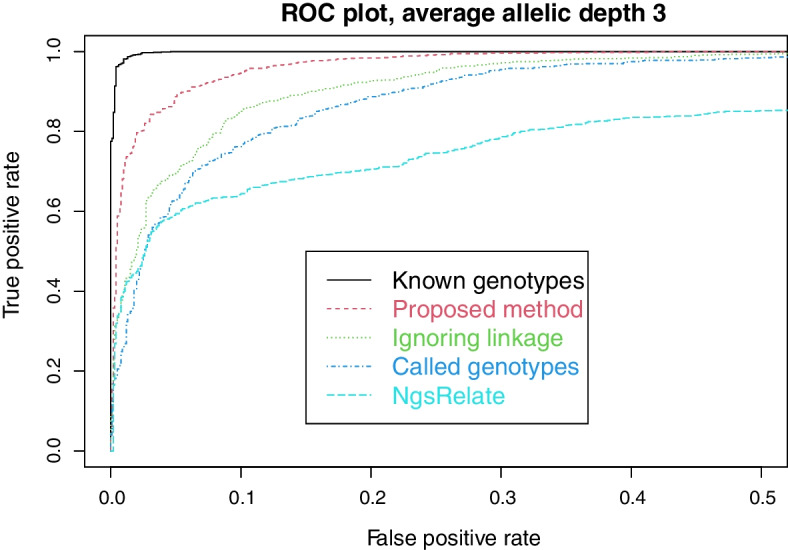
False positive and true positive rates for various methods, as the cutoff value changes

When the average allelic depth is reduced to 3, as in Fig. 4, we see that there is a considerable information loss in lcNGS data compared to data containing true genotypes. Nonetheless, our proposed method is able to resolve most cases. Note that calling the genotypes naturally leads to a much larger further loss of information at this allelic depth than at depth 10. These conclusions are also illustrated in Fig. 5.

Clearly, classification results for our proposed method will be worse for real data compared to results shown, as real data will not follow our stochastic model exactly. For example, setting the population fixation parameter $F_{st}$ to a value inappropriate for your population is likely to bias classification results. Regarding the $\gamma$ parameter, we simulated 100 cases with $\gamma =0.001$ and analyzed them with $\gamma =0.001$, $\gamma =0.00001$, and $\gamma =0$. ROC curves were indistinguishable for $\gamma =0.001$ and $\gamma =0.00001$, with insignificant differences to the case $\gamma =0$. Using inappropriate parameters *m* and *e* for the observational model is likely to impair results, but further study, involving real data, is needed to clarify these issues.

## Discussion

We describe a new model for likelihood computations with application to low count sequencing data. Essentially we report two advancements: An adaptation of the Lander-Green algorithm, including a general method to exploit symmetries for efficient computation, and a new proposed observational model for lcNGS data. The two advancements can be considered fairly independently.

The observational model can be used for likelihood computations for any purpose when lcNGS data is involved. It could be most useful when sample quality is not high. Several model improvements could be considered, such as involving quality information recorded for each locus in each sample. Clearly more analysis of real data is required, and we hope to return to this issue.

Our proposed algorithm for computations with linked loci can in principle be combined with any observational model. Indeed, our R code can take as input VCF-files where PL-fields indicate genotype likelihoods.

The general method should work if closely clustered SNPs are collected into “super-loci” with a correspondingly high number of possible alleles, ignoring the within-pedigree possibilities for crossovers between the clustered SNPs. This approach would be similar to [28]. However, there may also be other ways to include LD into the algorithm.

For now, one may choose between ignoring LD or extracting markers from the marker set such that LD is limited or absent from the panel where computations are performed. A pruning procedure may be used if whole genome data is used whereby, for instance, LD is computed for adjacent markers and removed if the degree of association is greater than some threshold.

Even STR markers could in principle be included. However, within-pedigree mutations would probably need to be ignored, which may not be an optimal choice.

An important job is to validate our approach on real cases, and to make it easy and accessible to use, and efficient to run. The current implementation in R is restricted to single core computations, however using parallelization is a fairly simple extension, for instance across chromosomes.

## Conclusion

Probabilistic pedigree inference may be done using low-coverage whole genome sequencing (lcNGS) data. We supply computational solutions by providing an R script. Computational time for a single likelihood ratio computation is about 1 min or less for the types of pedigrees investigated in this paper.

For high allelic depths and high-quality samples, computations may reliably be done by calling the genotype for each sample and locus based on read counts. For lower allelic depths or low-quality samples, reliable results may still be obtained, but require a probabilistic approach like the one we have implemented, rather than calling alleles.

Our method extends the Lander-Green algorithm to lcNGS data, and also implements use of a group of symmetries to speed up calculations with linked loci.

## Supplementary Information

**Additional file 1.** Appendix.

## Data Availability

The datasets used and/or analysed during the current study are available from the corresponding author on reasonable request.

## Footnotes

1 Note that an alternative parametrization could use a parameter $e'$ representing the probability of observing a different allele than the true one. Then $e'/3=e/4$.

2 In fact, we adjust each $L_i$ function with a factor so that its largest value is 1, to reduce problems with numerical underflow. This does not change the final likelihood ratio results.
