# Supplementary material for: Improved computations for relationship inference using low-coverage sequencing data
Source: BMC Bioinformatics. 2023 Mar 9;24:90. doi: 10.1186/s12859-023-05217-z (PMC9999603; doi:10.1186/s12859-023-05217-z)
Supplement: Supplementary file 1 — Additional file 1. Appendix. [file 12859_2023_5217_MOESM1_ESM.pdf]

Improved computations for relationship inference using low-coverage sequencing data

Petter Mostad, Andreas Tillmar, Daniel Kling

March 2023

Appendix

This appendix completes the theory of Section 2.6. For $r,r' \in \mathscr{A}$ and writing $r = (r_1,\ldots,r_K)$, $r' = (r'_1,\ldots,r'_K)$, define the Hamming distance

$$\|r - r'\| = \sum_{j=1}^{K} I(r_j \neq r'_j) \tag{1}$$

so that $\|r - r'\|$ counts the number of positions at which r and r' differ.Now define $\mathscr{S}$ as the set of permutations of $\mathscr{A}$ that leave such differences unchanged, i.e., for $g \in \mathscr{S}$ we have $\|g(r) - g(r')\| = \|r - r'\|$ for all $r,r' \in \mathscr{A}$. In fact, if we look at $\mathscr{A}$ as the corners of a hypercube in K dimensions, we see that $\mathscr{S}$ consists of all isometries of such cubes. Clearly $\mathscr{S}$ is a subgroup of the group of permutations of $\mathscr{A}$. For any $g \in \mathscr{S}$ we also have from Equation 8 that

$$T_i(g(r),g(r')) = p_i^{\|g(r)-g(r')\|}(1-p_i)^{K-\|g(r)-g(r')\|} = T_i(r,r') \tag{2}$$

for all $r,r' \in \mathscr{A}$.

Now, assume a pedigree has resulted in a particular function h partitioning the set of inheritance patterns into subsets that have the same IBD codes. Define the subgroup $\mathscr{S}_h \subseteq \mathscr{S}$ as those $g \in \mathscr{S}$ permuting values only within these subsets, i.e., we demand that $h(g(r)) = h(r)$ for all $r \in \mathscr{A}$. For $g \in \mathscr{S}_h$ we get that

$$\begin{aligned}
& \Pr(d_1,\ldots,d_i,g(r_i)) \\
= & \sum_{r_{i-1}} \Pr(d_1,\ldots,d_{i-1},r_{i-1}) \Pr(g(r_i) \mid r_{i-1}) \Pr(d_i \mid g(r_i)) \\
= & \sum_{r_{i-1}} \Pr(d_1,\ldots,d_{i-1},g(r_{i-1})) \Pr(g(r_i) \mid g(r_{i-1})) \Pr(d_i \mid g(r_i)) \\
= & \sum_{r_{i-1}} \Pr(d_1,\ldots,d_{i-1},g(r_{i-1})) T_i(g(r_{i-1}),g(r_i)) L_i(h(g(r_i))) \\
= & \sum_{r_{i-1}} \Pr(d_1,\ldots,d_{i-1},g(r_{i-1})) T_i(r_{i-1},r_i) L_i(h(r_i)).
\end{aligned} \tag{3}$$

From the equations above, and noting that $\Pr(r_1)$ is uniform, we get by induction that

$$\Pr(d_1,\dots,d_i,g(r_i)) = \Pr(d_1,\dots,d_i,r_i). \tag{4}$$

This proves Equation 13. Note that

$$\begin{aligned}
& \Pr(d_1,\dots,d_i,r_i=v_s) && (5)\\
= & \sum_{r_{i-1}} \Pr(d_1,\dots,d_{i-1},r_{i-1}) T_i(r_{i-1},v_s) L_i(h(v_s)) \\
= & \sum_{j=1}^{J}\sum_{k=1}^{n_j} \Pr(d_1,\dots,d_{i-1},r_{i-1}=v_{jk}) T_i(v_{jk},v_s) L_i(h(v_s)) \\
= & \sum_{j=1}^{J} \Pr(d_1,\dots,d_{i-1},r_{i-1}=v_j) \left(\sum_{k=1}^{n_j} T_i(v_{jk},v_s)\right) L_i(h(v_s))
\end{aligned}$$

which proves Equation 14.

To prove Equations 15 and 16, note that

$$\begin{aligned}
\sum_{k=1}^{n_j} T_i(v_{jk},v_s) &= \sum_{k=1}^{n_j} p_i^{\|v_{jk}-v_s\|}(1-p_i)^{K-\|v_{jk}-v_s\|} \\
&= \sum_{r=0}^{K} A_r(v_j,v_s) p_i^r (1-p_i)^{K-r} && (6)
\end{aligned}$$

where

$$A_r(v_j,v_s) = \sum_{k=1}^{n_j} I(\|v_{jk}-v_s\|=r). \tag{7}$$

Finding all symmetries

We start by developing some notation and theory. First, $\mathscr{A}$ is clearly a group, using element-wise addition modulo 2, and we may define the size of $r \in \mathscr{A}$ as $\|r\| = \sum_{i=1}^{K} r_i$. Also, any element $w \in \mathscr{A}$ may be viewed as an element in $\mathscr{S}$ by defining $w(r) = r + w$. Furthermore, consider the permutation group $\mathscr{G}$ on K integers, and let $z \in \mathscr{G}$ be coded as a permuted list of integers $z = (z_1,\dots,z_K)$. If $r = (r_1,\dots,r_K) \in \mathscr{A}$ we may define $z(r) = (r_{z_1}, r_{z_2},\dots,r_{z_K})$. Clearly $z \in \mathscr{S}$. In fact, let $g \in \mathscr{S}$ be such that $\|g(r)\| = \|r\|$ for all $r \in \mathscr{A}$. Then we see that $g = z$ for some $z \in \mathscr{G}$.

Note that in the group $\mathscr{S}$ elements z and w do not necessarily commute: We have $zw(r) = z(r+w) = z(r) + z(w) = z(w)(z(r))$ so $zw = z(w)z$. One may now show that any element in $\mathscr{S}$ may be written on the form wz, with $w \in \mathscr{A}$ and $z \in \mathscr{G}$, as follows: If $g \in \mathscr{S}$ satisfies $g(0) = 0$ then $\|g(r)\| = \|r\|$ for all $r \in \mathscr{A}$, and so $g = z$ for some $z \in \mathscr{G}$. For general g we get $(g(0)g)(0) = g(0)g(0) = 0$ so $g = g(0)g(0)g = g(0)z$ for some $z \in \mathscr{G}$.

Note that if $g_1 = w_1 z_1$ and $g_2 = w_2 z_2$ then $g_1 g_2 = w_1 z_1 w_2 z_2 = w_1 z_1(w_2) z_1 z_2 = (w_1 + z_1(w_2)) z_1 z_2$. Thus we have a way to store and multiply elements in $\mathscr{S}$.

Now assume a function h is given, and that we would like to find all elements in $\mathscr{S}_h$. We will write such elements on the form $g = wz$ with $z \in \mathscr{G}$ and $w \in \mathscr{A}$. Denote with $U_1,\dots,U_H$

the disjoint subsets that h divides $\mathscr{A}$ into. Let us further divide these into subsets

$$U_{i,j} = \{r \in U_j : \|r\| = i\} \tag{8}$$

for $i = 0,\ldots,K$, $j = 1,\ldots,H$. Note that if $wz \in \mathscr{S}_h$ we would need to have $z(U_j) = w(U_j)$ for all j. But as $\|z(r)\| = \|r\|$ for all r and all $z \in \mathscr{G}$, we must also have $z(U_{i,j}) = W_{i,j}$ for all i and j, where

$$W_{i,j} = \{r \in w(U_j) : \|r\| = i\}. \tag{9}$$

We describe our algorithm as follows: As a first step, compute $\#U_{i,j}$, i.e., the sizes of these sets. Then, for all $w \in \mathscr{A}$:

1. For $i = 0,\ldots,K$ and $j = 1,\ldots,H$ define $W_{i,j}$ according to Equation 9. Based on the above, we need to have $\#W_{i,j} = \#U_{i,j}$ for all i and j for w to be a viable candidate for a part of $g \in \mathscr{S}_h$ on the form $g = wz$. If indeed these counts are all the same:
2. For $g = wz$ to be in $\mathscr{S}_h$, z must map $U_{1,j}$ to $W_{1,j}$ for $j = 1,\ldots,H$. It is also clear that any $z \in \mathscr{G}$ is determined by its action on elements of size 1. Thus we may generate candidate values z by systematically going through the ways the sets $U_{1,j}$ can be mapped to the sets $W_{1,j}$. For each candidate z:
   (a) Check if z satisfies $wz(U_j) = U_j$ for $j = 1,\ldots,H$.
   (b) If it does, store $g = wz$ in a list of elements of $\mathscr{S}_h$.
